# The influence of personal communities in understanding avoidable emergency department attendance: qualitative study

**DOI:** 10.1186/s12913-020-05705-5

**Published:** 2020-09-21

**Authors:** Gemma McKenna, Anne Rogers, Sandra Walker, Catherine Pope

**Affiliations:** 1 Health Services Management Centre, School of Social Policy, University of Birmingham, Birmingham, B15 2RT UK; 2 NIHR CLAHRC Wessex, University of Southampton, Southampton, SO17 1BJ UK; 3 Nuffield Department of Primary Care Health Services, University of Oxford, Oxford, OX2 6GG UK

**Keywords:** Emergency department, Emergency care, Social networks, Inappropriate attendance, Qualitative methods, Help-seeking, Healthcare service

## Abstract

**Background:**

Use of emergency department (ED) care globally seems to be increasing at a faster rate than population growth (Baker, House of Commons Library. Accident and Emergency Statistics, Demand, Performance, 2017). In the UK there has been a reported 16% rise in emergency admissions over the past 5 years. Estimates that between 11 and 40% of ED attendances are non-urgent, with 11% of patients being discharged from the ED without treatment (NHS Digital 2017), and a further 44% require no follow-up treatment (NHS Digital, Hospital Accident and Emergency Activity 2016-17, 2019) is cited as evidence that these patients did not require this level of care. The solution to not using the most appropriate point in the system has traditionally been seen as a knowledge problem, requiring, improved sign-posting and information to enable people to self-manage or use health care management for minor ailments. However research about help-seeking behaviour suggests that the problem may not be an informational one. A considerable literature points to help seeking as a social process influenced by a range of contingencies and contextual factors including the way in which lay people influence health care utilisation (Giebel et al. BMJ Open 9:1, 2019). Personal communities comprise a variety of active and significant social ties which have potential to influence individual capacity to seek help. Here we extend and unpack further influencing decisions about seeking formal health care with reference to how they are shaped and informed by and within personal social networks.

**Methods:**

We undertook a personal network mapping and qualitative interview-based study to look at, problematize and understand attendance for non-urgent problems. We used network analysis and methods to map and characterise the personal communities of people seeking help from ED for minor ailments and semi-structured interviews with 40 people attending a single ED and associated GP hub providing equivalent care. Interviews were built around an ego network mapping activity and a topic guide structured to explore attender’s narratives about why they had visited the ED. This ego network activity uses a diagram consisting of three concentric circles (Fiori et al. J Gerontol B-Psychol 62: 322-30, 2007), representing closest social network members (in the centre) and those at further distance. Participants were initially presented with one of these diagrams and asked to write names of people or resources that had played a role in their attendance and the interviewer probed the interviewee to discuss the actions, input and value of the people and services that supported the visit to the ED.

**Results:**

We analysed number and type of network connections and undertook a thematic analysis to identify how imagined and actual network members and influences were implicated in ED attendance. The network maps created during the interviews were examined and a typology of networks was developed and used to distinguish different types of networks informed by our reading of the data, and a Network Typology Scoring Tool, a measure of frequency of contact and relationship type in networks.

**Conclusions:**

Our study suggests that faced with acute minor illness or injury people’s networks narrow: they do not (and perhaps cannot) mobilise their *imagined* care network because the resources or connections may not be there or are difficult to engage. In addition we identified important system drivers of behaviour, notably that these patients are often directed to the ED by ‘professional influencers’ including health services staff.

## Background

There has been a reported 16% rise in emergency admissions over the past 5 years and there are now an average of 3100 more emergency department (ED) (also referred to as Accident and Emergency/A&E) attendances each day. Use of emergency care is estimated to be increasing at a faster rate than population growth [1] and its increasing use is considered a global public health issue [2]. ‘Over-use’ of ED has been an enduring concern to health policy makers and service providers with emphasis directed to the problem of ‘avoidable’ ED attendance. It is suggested that between 11 to 40% of ED attendances are non-urgent, or ‘inappropriate’ [3, 4] and the fact that 11% of patients are discharged from the ED without treatment [5], and a further 44% require no follow-up treatment [3] is cited as evidence that these patients did not require this expensive care modality and would have been more appropriately dealt with in primary care. Previous research suggests that patients used experiential knowledge to discriminate between services they use and that emergency service use is recursively shaped by prior experience influenced by difficulties. Illustrated, for example, by navigating appointment systems in primary care [6, 7].

Elsewhere health services research has noted that rising ED demand is due to service fragmentation and patient confusion about where to seek help [8, 9]. A recent ethnographic study showed that ED use was influenced by experiential knowledge of poor integration between in-hours and out-of-hours care, and of the quality of care it was possible to access in primary care [7].

The solution to what has been construed as ‘inappropriate’ service use has traditionally been seen as a knowledge problem, requiring improved sign-posting and information to enable people to self-manage or more appropriately direct requests for health care to manage minor ailments. Successive health education campaigns, notably those designed to encourage people to use the NHS 111 triage phone service, [10] and promotion of self-care for minor illnesses [11] have been utilised to persuade patients to avoid burdening their local ED. However research about help-seeking behaviour suggests that the problem is not only an informational one. Rather help seeking has been shown to be a social process [12], and that decisions about seeking formal health care are made, shaped and informed by lay referral networks [7, 12, 13]. This focuses attention on the social environment surrounding patients, and asks us to examine how the system level and wider networks surrounding care affect the help-seeking processes [14]. Whilst the configuration of services surrounding ED have been the focus of recent research, showing how the supply side of health care shapes patient perceptions, the notion of lay referral points to the possible influence of significant others in seeking help. Here we extend this dual focus by exploring the role of personal communities and contacts implicating a wider set of social network members and relational work relevant to seeking help which might influence ED attendance.

Social networks are a collective and structural influence on individual social and health practices, behaviour and outcomes [15–17], and to secondary prevention for mental and physical health [18, 19]. Social network methods offer a perspective that places emphasis on the individual but draws attention away from individual behaviour towards an approach to understanding and analysing how interactions with other ties are connected to the mobilisation of resources. Social network analysis thus offers an opportunity to avoid the trap of victim blaming (and in this case the construction of the problem as one of ‘inappropriate attendance’), moving away from individualised responsibility and binary representations of ‘good’ and ‘bad’ forms of ED service use [20, 21] to include a focus on the influencing potential of network connections. In this paper we explore how a qualitative social network mapping approach might be applied to the problem of ‘inappropriate’ ED attendance for minor ailments. Our study mapped the personal networks of people attending ED for minor illness and injury in order to describe and explore their decision making around help-seeking. Using a social network mapping tool, developed as a visual method and technique for qualitative investigation, and face to face interviews, our analysis identified the social network and wider health system drivers of help seeking. We offer this to provide a more nuanced, social understanding of the problem of ED attendances.

### Understanding networks in relation to help seeking

Social network research considers the bridging and resource effects of social relationships, and pays attention to the relational connections and ties between actors that are embedded in personal communities or networks. Wasserman & Galaskiewicz [22] and Erickson’s [23] exploration of network members’ interaction draws on ideas about network membership to show how people obtain normative guidance by comparing their own attitudes with those of a reference group of similar others, and how decisions and behaviours are confirmed, reinforced, or rejected in these networks [24]. We view personal networks as a social set of connections and relationships, consisting of individual actors and interactions between network members and the resources they hold. Whilst previous research has identified the importance of ‘others’ (notably spouses and close kin) in influencing decisions to attend ED (for example, [25]) a network approach sees the wider social system, personal communities and their properties as a source of potential collective support [26]. Social networks extend beyond health professionals and close family to incorporate connections in the workplace, casual acquaintances, (weak ties) friends, and groups and resources that offer health and wellness benefits (or disbenefits). This collective understanding can be used to explore the ways in which network members operate as a potential set of resources which can be mobilised - sharing knowledge and experiences within a personal community and mobilising network relationships related to health and illness. For example Vassilev et al. [27] point to the involvement of a cognitive and grounded selection of social network members from a wide range of possible connections based on the potential pragmatic support that is needed in managing illness. These mechanisms are ***navigation***, the identification of who should be contacted to support decisions or to provide help/resources, ***negotiation*** within networks, and building ***collective efficacy*** (developing capacity aimed at successful management through shared efforts and objectives). These suggested mechanisms suggest a close interdependence between social and psychological processes to shed new light on ‘the problem’ of ED help-seeking.

## Method

The study formed part of a larger programme of research, exploring self-directed engagement and people’s relationship to support and health service use carried out by NIHR Collaboration for Leadership in Applied Health Research and Care (CLAHRC) Wessex.

The study was underpinned by a phenomenological approach using interviews in a single ED to look at, problematize and understand ‘inappropriate attendance’ in this setting. Interviews centred on personal mapping methods which were used to characterise and identify network members within personal communities of people seeking help from ED for minor ailments. We used semi-structured interviews, built around an ego network mapping activity. The topic guide was loosely structured to explore attender’s narratives about why they had visited the ED on this occasion. The topic guide was developed using an exploratory approach, focusing on adaptive questions around the instances that prompted participants’ decisions to attend the ED. The adaptive nature of the questions allowed the researcher to keep a mental note of interesting points to raise with the participants to support their reflections around decisions to access ED and importantly how they felt in this context. The topic guide was pilot tested on an initial site visit to the ED with participants that met the study inclusion criteria. The researcher asked for feedback from the participants at the end of the interview around the schedule and mapping tool. Following feedback the schedule was amended to include fuller explanation of the social mapping tool. The study materials, including the topic guide, information sheet, and consent form, were independently, expertly validated through the NHS Health Research Authority East of England NRES committee (REC 18/EE/0049, IRAS ID: 239514).

**Fig. 1:**
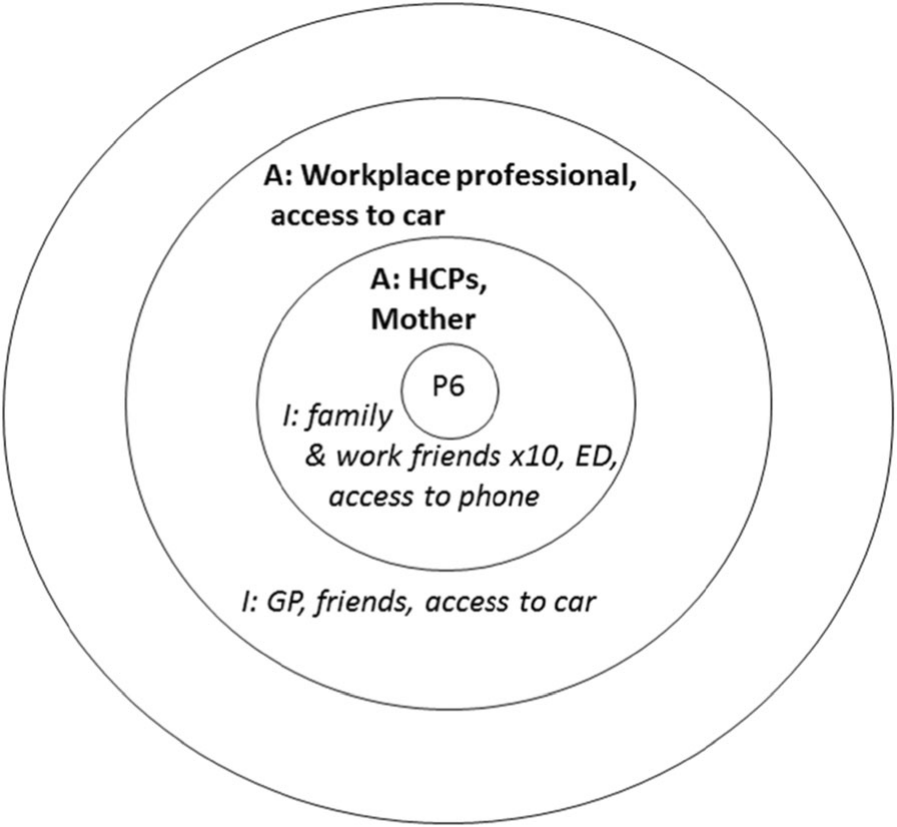
Example of participant’s **actual** network being very small and their *imagined* network being family supported

**Fig. 2:**
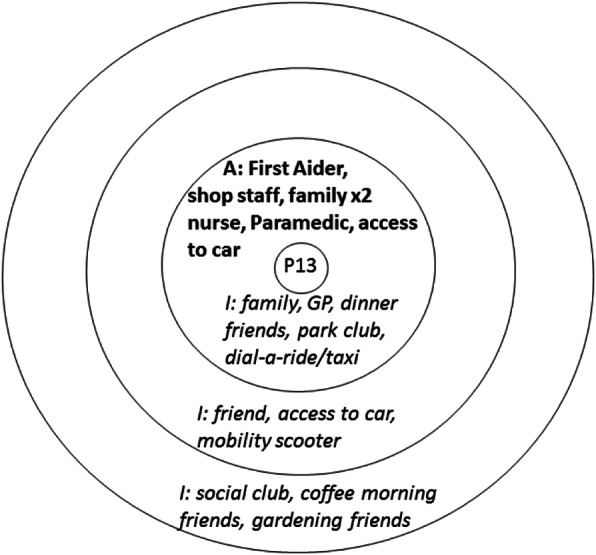
Example of participant’s **actual** network being very small and their *imagined network* being diverse

**Fig. 3:**
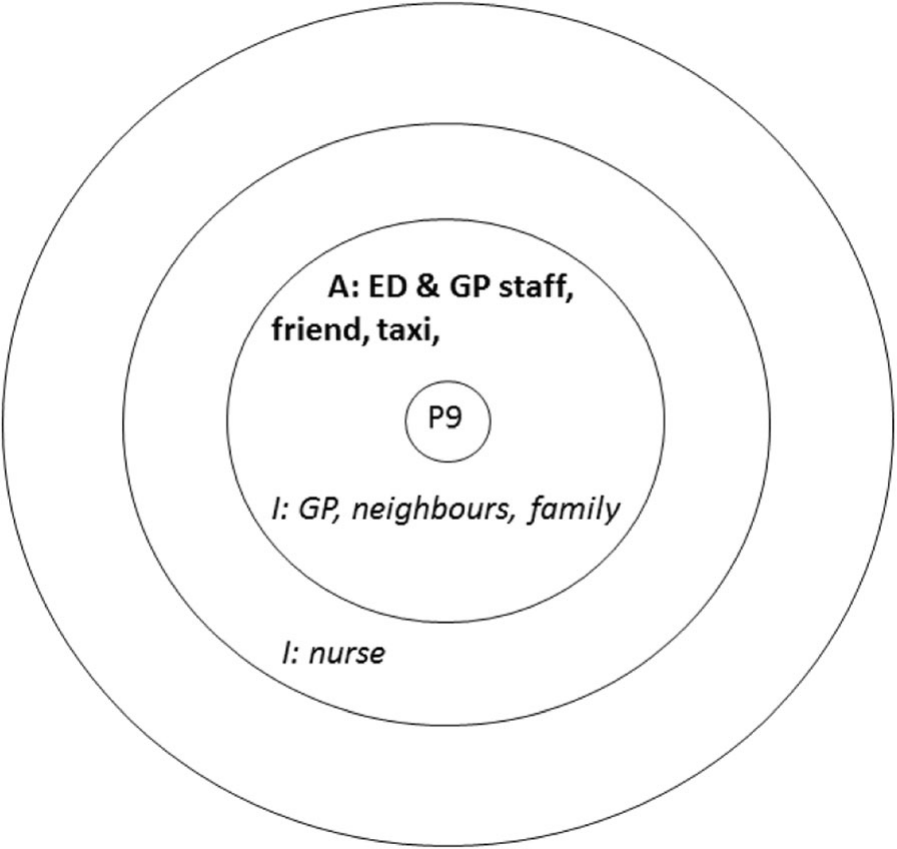
Example of participant’s **actual** and *imagined* networks as very small

The interview respondents were asked to complete a visual social network mapping exercise to map their personal social networks and to reflect on how these had informed their decision to attend (if at all). This approach enabled the participants to construct thoughts and share feelings that they did not know they knew or indeed felt. The social network activity uses a diagram consisting of three concentric circles [28], representing closest social network members (in the centre) and those at further distance. Participants were initially presented with one of these diagrams and asked to write names of people or resources that had played a role in their attendance. Using a think aloud method [29] the interviewer probed the interviewee to discuss the actions, input and value of the people and services that supported the visit to the ED. For example, identifying that a parent (written in the central circle) had encouraged them to seek help at the ED, and a neighbour (positioned in the next circle) had agreed to collect a child from school to enable this visit. Another map might show that the attender had first called their GP (written in the outermost circle), but had not been able to obtain an appointment. Once all the people and services had been identified the interviewee was asked to complete a second map showing what they ‘imagined’ an ideal network of support would look like. This enabled comparison between the real and idealised network use. This visual mapping exercise formed the basis for additional open ended questioning and probing for details about health beliefs, knowledge and previous use of health services, and the quality of social network relationships (see examples in Figs. 1, 2 and 3 in the analysis section).

Forty participants attending a single ED located in an acute Trust in South England were included in the study. The interviews were conducted by [GM and SW], both experienced female qualitative interviewers, one with a nursing background. The participants were sampled from those attending the minor treatment area in ED (*n* = 30) or a co-located GP hub providing equivalent care, located within the same hospital (*n* = 10). Participants were eligible for the study if they were over the age of 18, triaged on arrival as non-emergency, and deemed to have mental capacity to consent. We used convenience sampling, but attempted to ensure variation by conducting interviews at different times of day/night and on different days of the week. Those who met the inclusion criteria were initially identified by a nurse practitioner, and introduced to the researchers who invited them to participate and consequently obtained consent. Those who participated in the interviews were given a £10 voucher to thank them for their time. Ethical approval was granted from the NHS Health Research Authority East of England NRES committee (REC 18/EE/0049, IRAS ID: 239514).

Interviews were conducted in a screened off cubicle in the minor treatment area or in a private room/office nearby. Participants were mostly accompanied by a family member or partner. The interviews lasted between 25 and 45 min and were not audio recorded, in part because of the time constraints and the levels of ambient noise. The researchers introduced themselves at the start of the interviews, explained the reasons for doing the research, and how they hoped that the research outcomes would support understanding around the personal networks people use when accessing NHS services. The researchers took handwritten notes, near verbatim where possible, which were transcribed and anonymised after the interview, for analysis alongside the mapping diagrams which were retained.

### Analysis

A thematic analysis was undertaken to identify how imagined and practical network members and influences were implicated in ED attendance. The analysis moved from independent coding, immersion in the data through reading and re-reading the case histories, to grouping cases and data codes against themes, for example, focusing on a category of ‘vulnerable patients’ and examining data attached to this. AR and CP supported analysis in regular team data clinics where a description of the data coding tree was provided and emergent themes were discussed. Data coding and grouping were also revisited to enhance rigour. The network maps created during the interviews were examined and a typology of networks was developed and used to distinguish different types of networks. The development of the typology was informed by our reading of the data, but also by deductive use of Vassilev et al. [16] Network Typology Scoring Tool, a measure frequency of contact and relationship type in networks. We developed a typology shown in Table 1 which considers both the size of the network, its diversity, and the network relationships (e.g. familial, friendship) identified. The latter also included a category labelled ‘services supported’ which encompassed sources of support from formal health and or social care service providers, which might include a General Practitioner, a social care support worker or a Web based health information service.

**Table 1:** Typology of networks

Network type	Coding criteria
***Very diverse***	If family > = 20 **and** friends > = 15 **and** groups> = 2
**Diverse**	If family > = 20 **and** 0 < friends < 15 **and** groups> = 2 **OR** If 0 < family < 20 **and** friends > = 15 **and** groups> = 2 **OR** If family > = 20 **and** friends > = 15 **and** groups = 1
***Family and friend centred***	If family > = 20 **and** friends > = 15
***Friend centred***	If family < 20 **and** friends > = 15
***Family centred***	If family > = 20 **and** friends < 15
***Family and friend supported***	If 7 = < family < 20 **and** 5 = < friends< 15
***Friend supported***	If family < 7 **and** 5 = < friends< 15
***Family supported***	If 7 = < family < 20 **and** friends< 5
**Small**	If family < 7 **and** friends< 5 **and** overall score > = 8
**Very small**	If family < 7 **and** friends< 5 **and** overall score < 8

Table 2 summarises the characteristics of the sample. All those attending were considered by the triage nurse to have an injury or condition that did not require immediate treatment and the patient was therefore classified as capable of waiting to be seen. (For this reason we have not listed presenting condition in the table, however we have noted co-morbidities mentioned by the patient as these illustrate that not all those attending have a single illness or issue). We were able to include a good mix of gender and age reflecting attendances to the minors’ area of ED, but acknowledge that this group is less reflective of racial diversity (we did not have access to interpreter facilities). We have also highlighted participants who were Polish to reflect the large population within the geographical area and the lack of research associated with this group in the literature. The right hand column shows that many of the networks described were categorised as very small, and/or diverse, with several designated as family supported.

**Table 2:** Participant demographic information

	Gender	Age	Co-morbidities	Network typology ED / Imagined
1	Female	70’s-80’s	N/A	Family supported / Family supported
2	Male	20’s-30’s	Epilepsy	Very small / Family supported
3	Female	60’s-70’s	N/A	Very small / Diverse
4	Male	20’s-30’s	N/A	Very small / Diverse
5	Male	20’s-30’s	Mental ill-health	Very small / Diverse
6	Female	20’s-30’s	N/A	Very small / Family supported
7	Female	20’s-30’s	N/A	Very small / Very small
8	Male	40’s-50’s	N/A	Very small / Very small
9	Female	50’s-60’s	N/A	Very small / Very small
10	Male	20’s-30’s	N/A	Diverse / Diverse
11	Male	50’s-60’s	N/A	Very small / Very small
12	Male	20’s-30’s	unexplained symptoms/ low blood pressure	Family supported / Family supported
13	Male	90+	Elderly	Very small / Diverse
14	Male	50’s-60’s	Eye disease	Very small / Very small
15	Female	50’s-60’s	N/A	Family supported / Diverse
16	Female – Polish	30’s-40’s	N/A	Very small / Family supported
17	Male	40’s-50’s	N/A	Very small / Very small
18	Female	50’s-60’s	N/A	Very small / Very small
19	Female	20’s-30’s	N/A	Very small / Diverse
20	Female	20’s-30’s	N/A	Very small / Diverse
21	Male	50’s-60’s	N/A	Very small / Diverse
22	Female	70’s-80’s	N/A	Very small / Very small
23	Male – Polish	40’s-50’s	N/A	Family supported / Diverse
24	Female	70’s-80’s	N/A	Very small / Diverse
25	Female	70’s-80’s	N/A	Very small / Diverse
26	Female	40’s-50’s	Colorectal	Very small / very small -services supported
27	Female	30’s-40’s	N/A	Very small / Diverse
28	Female	60’s-70’s	N/A	Very small / Diverse
29	Male	60’s-70’s	N/A	Very small / Very small
30	Female	60’s-70’s	learning difficulties / diabetes / angina / leg ulcers	Very small-services supported / Very small services supported
31	Female	30’s-40’s	Mental ill-health	Very small-services supported / Very small- services supported
32	Female	50’s-60’s	N/A	Very small / Very small
33	Male	20’s-30’s	N/A	Very small / Very small
34	Female	20’s-30’s	Pregnant/ liver disease	Very small / Very small

This paper presents two core emergent themes from our analysis. The first was deductive, driven in part by our use of the two visual network mapping exercises and it describes and compares the real and imagined social networks. The second was more inductive, and developed from our interrogation of the ‘real’ network maps and the associated interview data exploring what may have prompted this ED attendance, and, as we show, shed some new light on the system drivers of this behaviour.

### Real and imagined networks and decisions to attend

Interviewees were encouraged to explain their present and **actual** network surrounding their decision making process before being asked to identify people and services they may or would like to have drawn on but did not (their *imagined* network), and to explain the reasons for these choices. The purpose of imagining the network was to explore alternative or complementary resources to ED attendance and to examine the gap between what was available in the here and now and what could potentially be available in future.

Our 40 respondents identified 200 network connections in their actual networks when attending the ED, 58 of whom were relatives, 18 friends/colleagues and 97 connections to the healthcare system, and 3 ‘other connections’ which included social groups. Most of the actual networks identified were very small, and family oriented. Imagined networks were larger, comprising 261 network connections and expanded the ‘other’ connections to include 36 sports clubs, support aids, internet and phone connection, pets and local authority support. Examples of these real and imagined networks are displayed in composite form in Figs. 1, 2 and 3 (with actual networks in bold).

In an acute minor episode participants’ networks appear to shrink and our interviewees described a highly individualistic approach to decision making. They often refused to think about mobilising their *imagined* care network because they ‘don’t want to bother people’, especially their own children and siblings. This resonates with other network studies [30], which suggest that reliance on the self in a critical moment of decision making is used to avoid perceptions of being a burden, and to manage threats to a core role identity (e.g. as a parent). Interviewees explained these decisions in the following ways:

*I have a son, but I don’t bother him with stuff, he doesn’t need to worry about his old man. So I don’t bother telling him stuff.* ***(P14 male)****.*

*I try not to tell her (sister) too much as she has her own health issues with her diabetes and she just worries about me and it makes her worse … well I don’t have any friends left now and I miss friendship. I do a slimming club [online] but I don’t go to the meetings so there’s not many people to tell.* ***(P30 female)****.*

*I’m not one to bother people. I would’ve liked to just see the GP and not tell anyone at all what had happened. I wouldn’t have told my brother out of choice because of the nature of the problem, it’s embarrassing (groin pain) and I didn’t want to worry anyone.* ***(P36 male).***

*We have friends in London who we could’ve talked to and our parents are nurse specialists so they could’ve helped but no one knows about the pregnancy and we didn’t want to worry anyone so we didn’t tell anybody and with the escalating pain we just decided to come straight to the ED.* ***(P37 female).***

These quotes highlight that family members are identified in imagined networks of care but are discounted as sources of support. Another interviewee explained that he used communication with family and friends initially, but then mobilised formal health care services:

*On the Saturday I didn’t feel too much pain; the impact was quite big but I thought I’d be ok. It was a bit upsetting though as I was just 30 yards from home. I got back home and put a cold compress on it and elevated it and took some painkillers. It started to swell and I was concerned something might be broken. I spoke to my friend and my mum and sister in Poland, who I am close to and thought about calling the GP. I run things past them. I thought it would be fine. But by today (3 day delay) it was very painful so I called the GP but the receptionist told me to that I needed to go to the A&E in case I needed an x-ray.* ***(P23 male)*.**

Our analysis confirmed that those attending the ED for minor illness and injury tended to have small networks. It was also clear that their decisions to attend the ED often discounted the input of network members, including close (inner circle) familial ties. When asked to imagine social networks that could support them with regard to their health care needs, they were able to suggest some wider membership, including services, that could potentially assist, but these were ruled out unsuitable or unavailable for the current circumstance/presentation.

### System drivers of ED attendance

Further scrutiny of our data suggested that while they seldom appeared in the inner circle of close relational ties in the network, health professionals and the wider health care system actors exerted considerable influence on help seeking. Health care professionals in personal networks can be valuable sources of health-related support in managing illness because of their perceived expertise and ability to provide access to medication, treatments and advice. However, at times they have been found to be less valued than their social status and level of training and expertise suggest [31]. In the interviews for our study health professionals and the services that employed them were characterised as being ambiguous in their actions and messages, risk averse, and offering diversionary strategies. As network members with authority and expertise they seemed instrumental in decisions to attend, and might, given the rhetoric about appropriateness, be expected to ‘push’ help-seeking towards primary and self-care and away from the ED. However legal and institutional imperatives appeared to prevent this: interviewees described how risk aversion appeared to favour management strategies that pushed minor cases to the ED. A number of interviewees were encouraged to attend by other health care providers, notably in primary care:

*I waited until Monday to call the GP for an appointment. I eventually got through to the nurse triage at my GP surgery. She said it wasn’t a GP appointment I needed and that I needed to call an ambulance, but I didn’t want an ambulance but she was quite militant and questioning and wouldn’t give me a doctor’s appointment. Getting past the receptionist and their telephone system is hard. … So I used 111 online [NHS Choices] for advice and it said to call an ambulance, well I didn’t want one, I thought ambulances are for RTAs [road traffic accidents] and things like that, but that was what the advice was so I called 999 for some advice, I was so cross though, I didn’t want to waste people’s time but they were really good and said I needed to get myself to A&E within the hour, so here I am. I think my doctor should’ve seen me, there’s a lot of passing the buck that goes on.* ***(P26 female).***

System or service influences on attendance were not always immediate or reactive, but could be cumulative over time, as concerns accrued and were presented to a professional or service network member. P26 quoted above identified few friends, her sister and social media for support, but explained that, living with a long term condition, she tended to ‘save all my problems up’ until she visited the [specialist] clinic. She said that the health professionals at this clinic ‘sort all of my problems out at once, it’s great’. This system behaviour encouraged reliance on specialist hospital care rather than General Practitioner services. The location of ED at the same hospital as these specialist facilities meant that it was seen as an extension of this specialist support. Another interviewee had several long-term conditions and learning difficulties and told a similar story about how the health care system ‘pushed’ a decision to attend ED. For her this choice was also impacted by transport constraints, again indicating limited network resources to support alternative help-seeking:

*I tried to book an appointment with the practice nurse to dress the ulcers on my legs but she said she didn’t have any appointments. I woke up and the bandage was half way down my leg, there was lots of pus coming out, green gungy stuff, so we thought it was an infection. We was going to go up the walk-in centre, but we didn’t know if they would just send us up here, and we have to rely on taxis, so could only afford one journey, so we came here.* ***(P30, female).***

The ED nurse who saw this patient subsequently described this example as ‘a medical emergency waiting to happen’ and suggested that the patient should have demanded to have a district nurse to come and visit her. Despite this professional view, and clear public health messages about appropriate attendance, it was clear in our data that relationships or ties to health service network members did not make it less likely that they would present at the ED. Indeed, in many cases patients knew that the ED was not the place where they should be, but they were advised to attend by other system-related network members, both individual health care professionals and the NHS 111 triage telephone service:

*I spoke to my counsellor about it, and then phoned the GP, but there weren’t any appointments. So we went to the nearest medical centre and saw a nurse who said it would be a very long wait but I couldn’t wait anymore, I was in too much pain. So I decided to come to A&E, my counsellor drove me. We then got sent here* ***(P31, female).***

In the morning the nurse encouraged me to call 111. They suggested that I go to A&E. ***(P19, female).***

*And I asked for an appointment about my knee, and the receptionist said I’d need an x-ray, so she told me to go to the Minor Injuries Unit, and then go back to the doctor, so here I am.* ***(P10, male).***

Eight of the ten participants interviewed in the GP hub had tried accessing help prior to making the decision to come to the ED. The remaining two were geographically closer to the ED, pointing to the role that spatial context plays in decision making: if health services are perceived as being near-by they will be used.

Other people in peripheral social network locations also played a part in encouraging ED attendance. Some of those who presented with minor injuries gave accounts that mentioned how workplace network members informed their decision-making. P22 provides an example of how workplace sanctioning of help-seeking was overlain with health care professional advice that led to an ED attendance. In this case, P22 was injured whilst in a supermarket:

*The first aider was very good and came to see me straight away, it was very painful. She advised me to go to the Minor Injuries Unit so I did. They told me it was probably a pulled muscle in my knee but told me to go to the A&E to get it checked just in case.* ***(P22 female).***

P6 worked in a school and was injured at work, and during her interview she explained that ‘they filled out the health and safety forms’. While a family member prompted her to attend the ED, this earlier sanctioning of the ‘seriousness’ of the injury played a role in her decision. Similarly, P11 injured himself at home but was aware of the need to get a certificate of fitness to work to comply with health and safety procedures in his work environment (on board a ship):

*I have to get this sorted out before going back to my ship...I have to get my medical certificate done, and because this happened off ship I have to have some evidence to show what is wrong.* ***(P11, male).***

Like P22 described above, P9 was injured in a public workplace (a library). The decision to attend ED was taken by a worker in this setting who called a taxi to take her to the ED, and P9 did not draw on other network members. Similarly P13 fell whilst in a cafe and was seen by a paramedic who happened to be there, and brought this elderly man to hospital.

Our thematic analysis suggests that people attending ED for minor illness and injury have small, or very small, networks. Two of our sample were supported by health and care services (one with mental illness (P31) and one with learning disability (P30)) but most identified close familial ties as the dominant relationship in their networks. Few of our respondents were able to imagine a wider network that they might call on in future, and they had clear reasons for not enrolling current network members in their decision to attend the ED on this occasion. One finding to emerge from our data, was that contra to the health education and policy rhetoric about appropriate attendance, it seems that attendees were often pushed by people outside these core familial and friendship networks to attend the ED for minor illnesses and injuries. Health professionals (including staff of the telephone triage service NHS 111) and/or workplace representatives (such as workplace first aiders, shopkeepers) encouraged them to attend the ED. A further finding, with reference to P11, highlights the requirement for individuals to produce medical certificates to employers to justify their work absence, perhaps contributing to ED attendance. Whilst in the UK there is an expectation that sickness notes come primarily from and are issued by primary care, this knowledge may not be shared by users of the system. This presents a significant bureaucratic issue and unnecessary source of concern for patients who receive contradictory public messages around not using health services but are still required to produce documents to validate any illness.

## Discussion

This study has provided further understanding of ED use and provides insights into hitherto unnoticed system influences through exploring the nature and negotiation of influence from personal communities. We have shown that using social network analysis provides unique insights into people’s help seeking behaviour when using the ED, showing how networks narrow due to lack of resources and difficulty engaging social connections. We have shown that despite the participants’ reluctance to attend ED, health care and other professionals often directed them to ED and away from primary care. These ‘professional influencers’ demonstrate a strong power dynamic as network members and are highly influential. These unique findings challenge existing debates around ‘inappropriate’ attendance at ED, calling instead for a broader view of networks and social features of help seeking.

Dodds et al. [32] in their analysis of network robustness suggest that in the face of ambiguity network members often exchange information with other ‘problem solvers’. Watts [33] argues that individuals organise their perception of the world in a hierarchical fashion. These models suggest that individuals might value people closest to them and place them in the middle of their networks. When accessing ED care this does not seem to be the case, indeed our analysis highlights deficits in existing social network resources and the role of more hidden agents. There is a gap between the number of actual network members considered to be of relevance in the decisions to attend ED and those expressed in people’s imagined networks of influence. These imagined network configurations derived from the perceptions of users of ED reflects evidence that the more diverse a network, in terms of resources and ties, the more likely they are to be of benefit to health outcomes [34]. More importantly, there is a qualitative difference in the network resources considered when making this decision; in practice people appear to be swayed more by ‘professional influencers’, health services staff and other workplace members, and draw less on familial network members or on imagined group and community resources. When deciding on appropriate action for minor ailments or injuries, health and work institutions and their representatives have more authority than close family bonds, or weak tie bridging capital. Referring back to Vassilev et al.’s [27] three mechanisms for mobilising support, we see that this reliance on peripheral professional influencers overrides the navigation and negotiation mechanisms for mobilising support and removes the possibility of collective efficacy: the fact that ‘the nurse/first aider/NHS 111 service told me to attend ED’ denies agency and legitimates attendance. If this is the case then the policy focus on the rationale and behaviour of the individual or close informal network members has been overplayed. Attention may need to be directed instead to health services and others in public spaces and work places. Other research suggests the ambiguity and uncertainty of managers making decisions in relation to the health and illness of their employees has the capacity to feed into taking actions which result in contacting the wrong part of the system at the wrong time [35].

There have been multiple efforts to educate the public to use the ‘right service’ for the ‘right purpose’ and numerous attempts at re-education aimed at the individual. From our analysis it is little wonder then that attempts to encourage self-care (for example by promoting the use of community or digital resources) or attempting to discourage ED attendance via health education messages fail. Whilst better information, including options for accessing alternative sources of help might be useful, the drivers in adjacent health and work systems warrant more attention. To date there has been little effort aimed at interventions in work place settings to offer occupationally-based means of dealing with minor ailments and injuries. Awareness in other parts of the health care system (e.g. primary care) of individuals’ network capacity and resources in seeking help is relevant to managing health and help seeking appropriately [36]. The management of minor ailments of workers has traditionally been seen as the purview of occupational health and changes and cuts to these services, as well as a risk-averse culture, may be a system driver that has not hitherto been recognised as affecting ED demand [37–39]. These concerns also apply to the health service itself. The risk averse practices of clinical and non-clinical staff may be pushing people towards the ED. This is especially true of NHS 111 where triage by non-clinical call handlers using decision support software appears to have increased, not decreased, ED and formal care use [31, 40].

We have suggested elsewhere [9] that health service fragmentation has increased confusion amongst the public about where to go to have their urgent health needs met. Minor illness and injury is a source of particular confusion. Longstanding familiarity with local hospital provision and the ED means it is well understood as a source of support, and people may prefer to attend ED, despite the long waiting times they may endure there. Additionally, because of the proximity of access to specialist professionals and services (such as x-ray) the ED remains a one-stop-shop and this may encourage attendance, rather than at other services with more restricted provision. There are contradictory findings about the impact of increasing primary care provision on ED attendance, but Behr and Diaz [41] found that people often tried to access their GP before attending ED and this finding resonates with some of the interview accounts in our analysis. Combined with the kinds of ‘pushes’ identified above it is easy to see why people feel that the use of ED for these conditions is appropriate.

## Conclusion

Our study suggests that faced with acute minor illness or injury people’s networks narrow: they do not (and perhaps cannot) mobilise their *imagined* care network because the resources or connections may not be there or are difficult to engage. Many of the people we interviewed who had attended the ED for such conditions understood the rhetoric and debate about appropriateness, but they were often directed to the ED by ‘professional influencers’ including health services staff. Health and other professional network members exert considerable power over decisions about help seeking for minor ailments and injuries. Using a network approach has allowed us to see actors and their actions as interdependent, and to see these wider network and structural influences on behaviour. We suggest that any intervention directed to reducing ED attendance for minor illness and injury, rather than perpetuating victim blaming by labelling attendance as ‘inappropriate’, should consider how different professionals could better influence help-seeking. This may require for example finding ways to shift the risk perceptions and advice offered by professional influencers. Likewise signposting to other services and self-management advice might be better directed to workplace first aiders, reception staff and the like, than at the individual patients. By attending to the wider social network and understanding the social features of this help seeking process we may open up possibilities for reconstructing patterns of ED attendance differently from current practice.

### Strengths and limitations

Whilst this study provides insights into how people negotiate attendance at the ED, there are some limitations. The sample were from a small geographical area in the South of England, and of limited cultural diversity. Despite this, the study also has strengths. The examination of the real and imagined networks of people managing their health needs in crisis provides a unique opportunity to further understand this area and to consider hitherto unnoticed service influences.

### Implications for practice and research

More research is needed to explore the relevance of other network-related mechanisms and composition for understanding and responding to endemic health system problems of need and demand. A social network intervention which can map an individual’s current support network, eliciting values and preferences for responding, and link people to accessible resources might be of assistance in urgent and emergency care settings [26]. Such an approach could alert professionals to real or absent opportunities for patients to engage in alternative courses of action to ED attendance and provide wider opportunities for utilising appropriate resources outside the ED.

## Supplementary information

**Additional file 1.** Interview guide. A short list of topic guide questions and a visual ‘ego’ diagram used during the semi-structured interviews.

## Data Availability

The datasets generated and/or analyzed during the current study are not publicly available. This is because participants did not consent for the data collected to be made public or shared with any other parties. Participants did consent to the use of anonymized quotes as used in the manuscript.

## Abbreviations

A&E: Accident and Emergency Department
ED: Emergency Department
GP: General Practitioner
NHS: National Health Service
