# Supplementary material for: The influence of personal communities in understanding avoidable emergency department attendance: qualitative study
Source: BMC Health Serv Res. 2020 Sep 21;20:887. doi: 10.1186/s12913-020-05705-5 (PMC7504825; doi:10.1186/s12913-020-05705-5)
Supplement: Supplementary file 1 — Additional file 1. Interview guide. A short list of topic guide questions and a visual ‘ego’ diagram used during the semi-structured interviews. [file 12913_2020_5705_MOESM1_ESM.docx]

*Research Ethics Committee Reference Number: 18/EE/0049*

### INTERVIEW SCHEDULE FOR SERVICE USER INTERVIEWS

**GENIE (Generating Engagement in Networks Involvement) Emergency Department Study**

OUTLINING THE STUDY

- This study is being carried out by the University of Southampton;
- Funded by the NIHR CLAHRC Wessex (National Institute for Health Research, Collaboration for Leadership in Applied Health Research and Care). This is an independent study - we do not work for the NHS;
- In this discussion we will ask you about your experiences of using emergency care in more detail and the social networks you currently use or could use to access A&E and/or alternative care, if appropriate, in the future.

**General background / context - participant's health and social circumstances**

- Family context (guardian or carer) / history / working patterns /age
- General health long-term condition / multiple conditions
- Location urban / rural

**Understanding attendance at the Emergency Department**

- Why are you here? Prior contact with ED before?
- What were the specific circumstances surrounding your emergency department visit and the factors which influenced decisions to attend?
- Who/what people and services supported in your emergency department attendance?

**Making choices**

- How did you choose to the emergency department (A&E) service/ **what sources of support did you use to make the decision / who did you contact? / who did you talk it through with?**
- Please reflect on any self-care or self-management steps you took prior to attending the emergency department (A&E) / **reasons for any delay**
- **What would help you to make a difference decision and use an alternative service to A&E / Advice? Text alerts? Leaflets? Adverts?**

**I need to know**

- **Who is it?**
- **What did they do?**
- **Who do they value?**
- **What does their network of influence look like?**

**GENIE social networking intervention tool within the emergency department:**

1. We want to find out about all of the people and services that supported you today to access care in the first diagram.
2. I’d also like you to think of an imaginary network of care, thinking about all of the people and services you might have drawn on today to help you manage your condition.

- Example of the diagram and further GENIE tool <http://genie-net.org/>)
